# Supplement to the Hospital

**Published:** 1888-04-07

**Authors:** 


The Hospital.

### THE NATIONAL PENSION FUND FOR NURSES.

*Founded, October* 1887.] PENSIONS ♦ SICKNESS ♦ ACCIDENT. [*Incorporated, February* 1888.

38, OLD JEWRY, CHEAPSIDE, LONDON, E.C.

PATRONESSES.

THE COUNTESS OF ROSEBERY. | LADY ROTHSCHILD.

VICE-PRESIDENTS.

THE EARL OF ABERDEEN.

LORD ROTHSCHILD.

SIR EDMUND HAY CURRIE.

HENRY HUCKS GIBBS, Esq.

E. A. HAMBRO, Esq.

JUNIUS S. MORGAN, Esq.

THE COUNCIL.

WALTER H. BURNS, Esq. (Messrs. J. S. Morgan and Co.), *Chairman.*

HENRY C. BURDETT, Esq. (The Founder), *Deputy Chairman.*

J. S. BRISTOWE, Esq., M.D., F.R.S., Senior Physician, St. Thomas's Hospital.

THOMAS BRYANT, Esq., F.R.C.S., Senior Surgeon, Guy's Hospital.

R. BRUDENELL-CARTER, Esq., F.R.C.S., Ophthalmic Surgeon, St. George's Hospital and National Hospital for the Paralysed.

F. C. CARR-GOMM, Esq., Chairman of the London Hospital.

PERCIVAL A. NAIRNE, Esq., Chairman Finance Committee, "Dreadnought" Seamen's Hospital.

EDWARD RAWLINGS, Esq. (late Messrs. C. I. Hambro and Son.)

MAJOR ROSS, M.P., Chairman of the Middlesex Hospital.

ALFRED CHARLES DE ROTHSCHILD, Esq. (Messrs. Rothschild and Sons.)

J. C. STEELE, Esq., M.D., Medical Superintendent, Guy's Hospital.

JOHN WATNEY, Esq. (Mercers' Hall, E.C.)

CLIFFORD WIGRAM, Esq. (Director of Bank of England.)

BANKERS—THE BANK OF ENGLAND AND ITS BRANCHES.

CONSULTING ACTUARY—GEORGE KING, Esq., F.F.A., F.I.A. SECRETARY—PHILLIP GROVE, Esq.

THE NATIONAL PENSION FUND is incorporated with the sanction of the Board of Trade, under the 23rd section of the Companies Act, 1867, and the Life Assurance Companies Acts, 1870 to 1872.

Its successful institution is due to the munificence of Lord Rothschild, Messrs. Henry H. Gibbs, E. A. Hambro, and Junius S. Morgan, who have deposited £5,000 each with the Accountant-General in Chancery, as security for the annuitants and policy-holders, and have consented that the dividends thereon shall be paid to the Bonus Fund until the accumulated premiums amount to £40,000.

Mr. Junius S. Morgan has given a second sum of £5,000 as the nucleus of a Bonus Fund; and others have promised subscriptions, which will raise it to about £6,000, so that the business of the National Pension Fund commences with an assured income of nearly £1,000 a year, available for the Bonus Fund. The constitution of the National Pension Fund provides for the continuous growth of the Bonus Fund, by means of annual subscriptions of two guineas and five guineas, or equivalent compounding fees of £25 and £50 respectively in one sum, from persons who are interested in nursing and nurses, and from authorities who train and employ nurses.

The National Pension Fund for Nurses is an institution the establishment of which has been long in contemplation. The founder, Mr. Burdett, has laboured diligently for many years in order to ensure that the fund should be so organised as to meet the wishes and requirements of all whom it is desired to benefit. Its chief object is to afford to nurses a means of providing, at the lowest possible cost to themselves, an allowance during incapacity for work caused by sickness or accident, and a certain income during their declining years. This object will be carried out by receiving and investing such fixed periodical sums as those who join the fund can afford, and by supplementing these sums by a bonus fund, created and maintained by those interested in nurses and nursing institutions. The tables given on the next page have been so arranged that, for a quarterly payment, the amount of which depends on the age at joining the fund, a minimum pension is guaranteed; and it is hoped that, by means of the Bonus Fund, such pension will be materially increased. A nurse of twenty-five years of age, with a salary of £25 per annum, who pays into the fund £3 5*s*. each year, or 5*s*. 6*d*. a month, will be able to ensure 10*s*. a week, whether totally incapacitated by sickness or accident at any time up to the age of sixty, and after sixty a minimum pension of £15 a year for the rest of her life. Again, a nurse of twenty-five years of age who pays to the fund £2 10*s*. a year, or 4*s*. 2*d*. a month, will be able to ensure a minimum pension of £15 a year after sixty years of age for the rest of her life. If she desires to secure the return of the money she may have paid to the fund before the pension has begun to be paid, she will have to pay £3 6*s*. 8*d*. a year, or 5*s*. 7*d*. a month. Should any nurse aged twenty-five desire to ensure an allowance of 10*s*. a week during the time she may be permanently disabled by sickness or accident, she may do it by a further payment of 15*s*. a year, or 1*s*. 3*d*. per month, making £3 5*s*. a year, or 5*s*. 5*d*. per month, in the first instance, and £4 1*s*. 8*d*. a year, or 6*s*. 10*d*. per month in the second. The pension of £15 is actually guaranteed, and the hope is that such pension will be materially increased by the Bonus Fund.

Tables I and II on the next page show the rates of contribution to secure minimum pensions commencing at age fifty, fifty-five, or sixty, these pensions to be increased from time to time by means of the Bonus Fund. Tables III and IV show the rates of contribution to secure a sick allowance combined with a pension. Table V is specially adapted to the cases of nurses working in hospitals who desire to seek lighter employment on attaining fifty years of age. Attention is directed to the explanatory headings of the tables.

To meet the cases of nurses wishing to pay a single sum to the fund in order to secure a pension, or of patients who might be desirous of making a provision for their nurses, Table VI has been added.

The cases of nurses who are upwards of fifty years of age, who may desire to invest their savings in the fund will be considered on written application to the secretary, accompanied by a statement showing their age next birthday, the amount of their savings available, the amount of pension they desire to secure, and the age at which the latter is to be payable.

The Council reserve to themselves the right from time to time to alter or vary the rules and tables laid down in this prospectus, if in their opinion the working of the fund, or any other circumstances should render it desirable to do so.

A form of proposal may be obtained on application, which should be filled in and signed by the applicant seeking admission to the fund, who must then forward it direct to the secretary, Mr. Philip Grove, 38, Old Jewry, London, E.C.

**Unless one thousand annuity policies are applied for by 1st January, 1890, the Guarantee and Bonus Funds will be returned to the donors and the fund wound up.**

### I.—Pensions only at 50, 55, or 60 (contributions not returnable).

(*Contributions not returnable under any circumstances.*)

Showing the Quarterly Contributions for

Pensions of £15 per annum, £22 10s. per annum, and £30 per annum, to commence at ages 50, 55, or 60.

FOR MATRONS, SISTERS, AND NURSES.

| Age next birthday. | Pension £15. | | | Pension £22 10s. | | | Pension £30. | | |
|---|---|---|---|---|---|---|---|---|---|
| | 50 | 55 | 60* | 50 | 55 | 60† | 50 | 55 | 60‡ |
| | £ s. d. | £ s. d. | £ s. d. | £ s. d. | £ s. d. | £ s. d. | £ s. d. | £ s. d. | £ s. d. |
| 20 | 1 2 11 | 0 15 0 | 0 9 8 | 1 14 5 | 1 2 6 | 0 14 6 | 2 5 10 | 1 10 0 | 0 19 4 |
| 21 | 1 4 3 | 0 15 11 | 0 10 2 | 1 16 5 | 1 3 9 | 0 15 3 | 2 8 6 | 1 11 10 | 1 0 4 |
| 22 | 1 5 8 | 0 16 10 | 0 10 8 | 1 18 6 | 1 5 3 | 0 16 0 | 2 11 4 | 1 13 8 | 1 1 4 |
| 23 | 1 7 3 | 0 17 9 | 0 11 3 | 2 0 11 | 1 6 8 | 0 16 11 | 2 14 6 | 1 15 6 | 1 2 2 |
| 24 | 1 8 11 | 0 18 9 | 0 11 11 | 2 3 5 | 1 8 2 | 0 17 10 | 2 17 10 | 1 17 6 | 1 3 10 |
| 25 | 1 10 5 | 0 19 9 | 0 12 6 | 2 5 8 | 1 9 8 | 0 18 9 | 3 0 10 | 1 19 6 | 1 5 0 |
| 26 | 1 12 9 | 1 1 0 | 0 13 2 | 2 9 2 | 1 11 6 | 0 19 9 | 3 5 6 | 2 2 0 | 1 6 4 |
| 27 | 1 15 0 | 1 2 3 | 0 13 11 | 2 12 6 | 1 13 5 | 1 0 11 | 3 10 0 | 2 4 6 | 1 7 10 |
| 28 | 1 17 5 | 1 3 8 | 0 14 8 | 2 10 2 | 1 15 6 | 1 2 0 | 3 14 10 | 2 7 4 | 1 9 4 |
| 29 | 2 0 2 | 1 5 2 | 0 15 6 | 3 0 3 | 1 17 9 | 1 3 3 | 4 0 4 | 2 10 4 | 1 11 0 |
| 30 | 2 3 0 | 1 6 8 | 0 16 5 | 3 4 6 | 2 0 0 | 1 4 8 | 4 6 0 | 2 13 4 | 1 12 10 |
| 31 | 2 6 3 | 1 8 6 | 0 17 5 | 3 9 5 | 2 2 9 | 1 6 2 | 4 12 6 | 2 17 0 | 1 14 0 |
| 32 | 2 10 0 | 1 10 5 | 0 18 6 | 3 15 0 | 2 5 8 | 1 7 9 | 5 0 0 | 3 0 10 | 1 17 0 |
| 33 | 2 14 2 | 1 12 6 | 0 19 8 | 4 1 3 | 2 8 9 | 1 9 6 | 5 8 4 | 3 5 0 | 1 19 4 |
| 34 | 2 18 11 | 1 14 11 | 1 0 11 | 4 8 5 | 2 12 5 | 1 11 5 | 5 17 10 | 3 9 10 | 2 1 10 |
| 35 | 3 4 2 | 1 17 6 | 1 2 3 | 4 16 3 | 2 16 3 | 1 13 5 | 6 8 4 | 3 15 0 | 2 4 6 |
| 36 | 3 10 6 | 2 0 5 | 1 3 9 | 5 5 9 | 3 0 8 | 1 15 8 | 7 1 0 | 4 0 10 | 2 7 6 |
| 37 | 3 17 5 | 2 4 0 | 1 5 5 | 5 16 2 | 3 6 0 | 1 18 2 | 7 14 10 | 4 8 0 | 2 10 10 |
| 38 | 4 5 9 | 2 7 3 | 1 7 2 | 6 8 8 | 3 10 11 | 2 0 9 | 8 11 6 | 4 14 6 | 2 14 4 |
| 39 | 4 15 8 | 2 12 2 | 1 9 2 | 7 3 6 | 3 18 3 | 2 3 9 | 9 11 4 | 5 4 4 | 2 18 4 |
| 40 | 5 7 6 | 2 16 2 | 1 11 5 | 8 1 3 | 4 4 3 | 2 7 2 | 10 15 0 | 5 12 4 | 3 2 10 |
| 41 | | 3 1 6 | 1 13 11 | | 4 12 3 | 2 10 11 | | 6 3 0 | 3 7 10 |
| 42 | | 3 7 9 | 1 16 8 | | 5 1 8 | 2 15 0 | | 6 15 6 | 3 13 4 |
| 43 | | 3 15 2 | 1 19 9 | | 5 12 9 | 2 19 8 | | 7 10 4 | 3 19 6 |
| 44 | | 4 3 11 | 2 3 3 | | 6 5 11 | 3 4 11 | | 8 7 10 | 4 6 6 |
| 45 | | 4 14 5 | 2 7 3 | | 7 1 8 | 3 10 11 | | 9 8 10 | 4 14 6 |
| 46 | | | 2 11 11 | | | 3 17 11 | | | 5 3 10 |
| 47 | | | 2 17 3 | | | 4 5 11 | | | 5 14 6 |
| 48 | | | 3 3 6 | | | 4 14 8 | | | 6 7 0 |
| 49 | | | 3 11 0 | | | 5 6 6 | | | 7 2 0 |
| 50 | | | 4 0 2 | | | 6 0 3 | | | 8 0 4 |

Anyone may enter for higher rates of pension, particulars as to which may be obtained on application to the Secretary, stating amount desired.

* In the case of those entering for a pension at 60 years of age, it is hoped that the Bonus additions will bring the Pension up to £26 per annum.

† In the case of those entering for a pension at 60 years of age, it is hoped that the Bonus additions will bring the Pension up to £30 per annum.

‡ In the case of those entering for a pension at 60 years of age, it is hoped that the Bonus additions will bring the Pension up to £40 per annum.

### II.—Pensions only at 50, 55, or 60 (contributions returnable).

(*The Contributions to be returned in the event of death or withdrawal after two years, and before the Pension is entered upon.*)

Showing the Quarterly Contributions for

Pensions of £15 per annum, £22 10s. per annum, and £30 per annum, to commence at ages 50, 55, or 60.

FOR MATRONS, SISTERS, AND NURSES.

| Age next birthday. | Pension £15. | | | Pension £22 10s. | | | Pension £30. | | |
|---|---|---|---|---|---|---|---|---|---|
| | 50 | 55 | 60* | 50 | 55 | 60† | 50 | 55 | 60‡ |
| | £ s. d. | £ s. d. | £ s. d. | £ s. d. | £ s. d. | £ s. d. | £ s. d. | £ s. d. | £ s. d. |
| 20 | 1 7 6 | 0 19 3 | 0 13 5 | 2 1 3 | 1 8 11 | 1 0 2 | 2 15 0 | 1 18 6 | 1 6 10 |
| 21 | 1 8 11 | 1 0 2 | 0 13 11 | 2 3 5 | 1 10 3 | 1 0 11 | 2 17 10 | 2 0 4 | 1 7 10 |
| 22 | 1 10 5 | 1 1 2 | 0 14 6 | 2 5 8 | 1 11 9 | 1 1 9 | 3 0 10 | 2 2 4 | 1 9 0 |
| 23 | 1 12 2 | 1 2 2 | 0 15 2 | 2 8 3 | 1 13 3 | 1 2 9 | 3 4 4 | 2 4 4 | 1 10 4 |
| 24 | 1 13 11 | 1 3 3 | 0 15 11 | 2 10 5 | 1 14 11 | 1 3 11 | 3 7 10 | 2 6 6 | 1 11 10 |
| 25 | 1 15 11 | 1 4 5 | 0 16 8 | 2 13 11 | 1 16 8 | 1 5 0 | 3 11 10 | 2 8 10 | 1 13 4 |
| 26 | 1 18 0 | 1 5 8 | 0 17 5 | 2 17 0 | 1 18 6 | 1 6 2 | 3 16 0 | 2 11 4 | 1 14 10 |
| 27 | 2 0 3 | 1 7 0 | 0 18 3 | 3 0 5 | 2 0 6 | 1 7 5 | 4 0 6 | 2 14 0 | 1 16 6 |
| 28 | 2 2 9 | 1 8 6 | 0 19 2 | 3 4 2 | 2 2 9 | 1 8 9 | 4 5 6 | 2 17 0 | 1 18 4 |
| 29 | 2 5 6 | 1 10 2 | 1 0 2 | 3 8 3 | 2 5 3 | 1 10 3 | 4 11 0 | 3 0 4 | 2 0 4 |
| 30 | 2 8 8 | 1 11 11 | 1 1 2 | 3 13 0 | 2 7 11 | 1 11 9 | 4 17 4 | 3 3 10 | 2 2 4 |
| 31 | 2 12 0 | 1 13 9 | 1 2 3 | 3 18 0 | 2 10 8 | 1 13 5 | 5 4 0 | 3 7 6 | 2 4 6 |
| 32 | 2 15 9 | 1 15 9 | 1 3 5 | 4 3 7 | 2 13 8 | 1 15 2 | 5 11 6 | 3 11 6 | 2 6 10 |
| 33 | 3 0 0 | 1 18 0 | 1 4 8 | 4 10 0 | 2 17 0 | 1 17 0 | 6 0 0 | 3 16 0 | 2 9 4 |
| 34 | 3 4 11 | 2 0 6 | 1 6 0 | 4 17 5 | 3 0 9 | 1 19 0 | 6 9 10 | 4 1 0 | 2 12 0 |
| 35 | 3 10 3 | 2 3 3 | 1 7 6 | 5 5 5 | 3 4 11 | 2 1 3 | 7 0 6 | 4 6 6 | 2 15 0 |
| 36 | 3 16 6 | 2 6 3 | 1 9 3 | 5 14 9 | 3 9 5 | 2 3 10 | 7 13 0 | 4 12 6 | 2 18 6 |
| 37 | 4 3 8 | 2 9 8 | 1 11 0 | 6 5 6 | 3 14 6 | 2 6 6 | 8 7 4 | 4 19 4 | 3 2 0 |
| 38 | 4 12 0 | 2 13 5 | 1 12 11 | 6 18 0 | 4 0 2 | 2 9 5 | 9 4 0 | 5 6 10 | 3 5 10 |
| 39 | 5 2 0 | 2 17 8 | 1 15 0 | 7 13 0 | 4 6 6 | 2 12 6 | 10 4 0 | 5 15 4 | 3 10 0 |
| 40 | 5 14 0 | 3 2 5 | 1 17 5 | 8 11 0 | 4 13 8 | 2 16 2 | 11 8 0 | 6 4 10 | 3 14 10 |
| 41 | | 3 7 11 | 2 0 0 | | 5 1 11 | 3 0 0 | | 6 15 10 | 4 0 0 |
| 42 | | 3 14 5 | 2 2 11 | | 5 11 8 | 3 4 5 | | 7 8 10 | 4 5 10 |
| 43 | | 4 1 9 | 2 6 2 | | 6 2 8 | 3 9 3 | | 8 3 6 | 4 12 4 |
| 44 | | 4 10 8 | 2 9 11 | | 6 16 0 | 3 14 10 | | 9 1 4 | 4 19 10 |
| 45 | | 5 1 3 | 2 14 0 | | 7 11 11 | 4 1 2 | | 10 2 6 | 5 8 0 |
| 46 | | | 2 18 8 | | | 4 8 2 | | | 5 17 4 |
| 47 | | | 3 4 5 | | | 4 16 8 | | | 6 8 10 |
| 48 | | | 3 10 9 | | | 5 6 2 | | | 7 1 6 |
| 49 | | | 3 18 5 | | | 5 17 8 | | | 7 16 10 |
| 50 | | | 4 7 8 | | | 6 11 6 | | | 8 15 4 |

Anyone may enter for higher rates of Pension, particulars as to which may be obtained on application to the Secretary stating amount desired.

* In the case of those entering for a pension at 60 years of age, it is hoped that the Bonus additions will bring the Pension up to £26 per annum.

† In the case of those entering for a pension at 60 years of age, it is hoped that the Bonus additions will bring the Pension up to £30 per annum.

‡ In the case of those entering for a pension at 60 years of age, it is hoped that the Bonus additions will bring the Pension up to £40 per annum.

### III.—Sick Pay and Pension (not returnable).

*Contributions not returnable under any circumstances.*

Showing the Quarterly Contributions to secure a
Sick Allowance of 10s. per week, with a Pension of £15 per ann.
Sick Allowance of 15s. per week, with a Pension of £22 10s. per ann.
Sick Allowance of 20s. per week, with a Pension of £30 per ann.
The Contributions and the Sick Allowance in each case to cease, and the Pension to commence at age 60.

FOR MATRONS, SISTERS, AND NURSES.

| Age next Birthday. | Sick Allowance, 10s. per week, Pension, £15.* | Sick Allowance, 15s. per week. Pension, £22 10s. † | Sick Allowance, 20s. per week. Pension, £30.‡ |
|---|---|---|---|
| | £ s. d. | £ s. d. | £ s. d. |
| 20 | 0 13 0 | 0 19 6 | 1 6 0 |
| 21 | 0 13 7 | 1 0 5 | 1 7 2 |
| 22 | 0 14 2 | 1 1 3 | 1 8 4 |
| 23 | 0 14 10 | 1 2 4 | 1 9 8 |
| 24 | 0 15 7 | 1 3 4 | 1 11 2 |
| 25 | 0 16 3 | 1 4 5 | 1 12 6 |
| 26 | 0 17 0 | 1 5 7 | 1 14 0 |
| 27 | 0 17 10 | 1 6 10 | 1 15 8 |
| 28 | 0 18 8 | 1 8 0 | 1 17 4 |
| 29 | 0 19 7 | 1 9 5 | 1 19 2 |
| 30 | 1 0 7 | 1 10 11 | 2 1 2 |
| 31 | 1 1 9 | 1 12 7 | 2 3 5 |
| 32 | 1 2 11 | 1 14 5 | 2 5 10 |
| 33 | 1 4 2 | 1 16 4 | 2 8 4 |
| 34 | 1 5 7 | 1 18 5 | 2 11 1 |
| 35 | 1 7 0 | 2 0 7 | 2 14 2 |
| 36 | 1 8 8 | 2 3 0 | 2 17 3 |
| 37 | 1 10 5 | 2 5 8 | 3 0 10 |
| 38 | 1 12 4 | 2 8 5 | 3 4 8 |
| 39 | 1 14 6 | 2 11 8 | 3 9 0 |
| 40 | 1 16 11 | 2 15 4 | 3 13 10 |
| 41 | 1 19 7 | 2 19 4 | 3 19 2 |
| 42 | 2 2 7 | 3 3 9 | 4 5 1 |
| 43 | 2 5 10 | 3 8 9 | 4 11 8 |
| 44 | 2 9 7 | 3 14 4 | 4 19 1 |
| 45 | 2 13 9 | 4 0 7 | 5 7 6 |
| 46 | 2 18 8 | 4 7 11 | 5 17 4 |
| 47 | 3 4 3 | 4 16 4 | 6 8 6 |
| 48 | 3 10 9 | 5 5 5 | 7 1 6 |
| 49 | 3 18 7 | 5 17 9 | 7 17 1 |
| 50 | 4 8 0 | 6 12 0 | 8 16 0 |

* It is hoped the Bonus additions will bring the Pension up to £26 per annum. † It is hoped the Bonus additions will bring the Pension up to £30 per annum. ‡ It is hoped the Bonus additions will bring the Pension up to £40 per annum.

### IV.—Sick Pay and Pension (returnable).

*The whole of the Contributions for the Pension benefit to be returned in the event of the death or withdrawal after two years, and before the Pension is entered upon.*

Showing the Quarterly Contributions to secure a
Sick Allowance of 10s. per week, with a Pension of £15 per ann.
Sick Allowance of 15s. per week, with a Pension of £22 10s. per ann.
Sick Allowance of 20s. per week, with a Pension of £30 per ann.
The Contributions and the Sick Allowance in each case to cease, and the Pension to commence at age 60.

FOR MATRONS, SISTERS, AND NURSES.

| Age next Birthday. | Sick Allowance, 10s. per week. Pension, £15.* | Sick Allowance, 15s. per week. Pension, £22 10s.† | Sick Allowance, 20s. per week. Pension, £30.‡ |
|---|---|---|---|
| | £ s. d. | £ s. d. | £ s. d. |
| 20 | 0 16 9 | 1 5 2 | 1 13 6 |
| 21 | 0 17 4 | 1 6 1 | 1 14 8 |
| 22 | 0 18 0 | 1 7 0 | 1 16 0 |
| 23 | 0 18 9 | 1 8 2 | 1 17 6 |
| 24 | 0 19 7 | 1 9 5 | 1 19 2 |
| 25 | 1 0 5 | 1 10 8 | 2 0 10 |
| 26 | 1 1 3 | 1 12 0 | 2 2 6 |
| 27 | 1 2 2 | 1 13 4 | 2 4 4 |
| 28 | 1 3 2 | 1 14 9 | 2 6 4 |
| 29 | 1 4 3 | 1 16 5 | 2 8 6 |
| 30 | 1 5 4 | 1 18 0 | 2 10 8 |
| 31 | 1 6 7 | 1 19 10 | 2 13 1 |
| 32 | 1 7 10 | 2 1 10 | 2 15 8 |
| 33 | 1 9 2 | 2 3 10 | 2 18 4 |
| 34 | 1 10 8 | 2 6 0 | 3 1 3 |
| 35 | 1 12 3 | 2 8 5 | 3 4 6 |
| 36 | 1 14 2 | 2 11 2 | 3 8 3 |
| 37 | 1 16 0 | 2 14 0 | 3 12 0 |
| 38 | 1 18 1 | 2 17 1 | 3 16 2 |
| 39 | 2 0 4 | 3 0 5 | 4 0 8 |
| 40 | 2 2 11 | 3 4 4 | 4 5 10 |
| 41 | 2 5 8 | 3 8 5 | 4 11 4 |
| 42 | 2 8 10 | 3 13 2 | 4 17 7 |
| 43 | 2 12 3 | 3 18 4 | 5 4 6 |
| 44 | 2 16 3 | 4 4 3 | 5 12 5 |
| 45 | 3 0 6 | 4 10 10 | 6 1 0 |
| 46 | 3 5 5 | 4 18 2 | 6 10 10 |
| 47 | 3 11 5 | 5 7 1 | 7 2 10 |
| 48 | 3 18 0 | 5 16 11 | 7 16 0 |
| 49 | 4 6 0 | 6 8 11 | 8 11 11 |
| 50 | 4 15 6 | 7 3 3 | 9 11 0 |

Anyone may enter for higher rates of pension, particulars as to which may be obtained on application to the Secretary, stating amount desired.

* It is hoped the Bonus additions will bring the Pension up to £26 per annum. † It is hoped the Bonus additions will bring the Pension up to £30 per annum. ‡ It is hoped the Bonus additions will bring the Pension up to £40 per annum.

### V.—Pensions of £10 at 50, increasing to a minimum of £15 at 60 (returnable or not returnable).

Showing the Quarterly Contributions to secure a
Pension of £10, to commence at age 50, and a further Pension of £5, making £15 per annum, to commence at age 60.
*Contributions to cease at age 50.*

FOR MATRONS, SISTERS, AND NURSES.

| Age. | Quarterly Contributions returnable if death occurs before age 50. | Quarterly Contributions not returnable. |
|---|---|---|
| | £ s. d. | £ s. d. |
| 20 | 1 2 9 | 0 19 0 |
| 21 | 1 4 0 | 1 0 1 |
| 22 | 1 5 3 | 1 1 3 |
| 23 | 1 6 8 | 1 2 7 |
| 24 | 1 8 2 | 1 4 0 |
| 25 | 1 9 9 | 1 5 6 |
| 26 | 1 11 6 | 1 7 2 |
| 27 | 1 13 5 | 1 9 0 |
| 28 | 1 15 6 | 1 11 0 |
| 29 | 1 17 9 | 1 13 3 |
| 30 | 2 0 4 | 1 15 8 |
| 31 | 2 3 2 | 1 18 5 |
| 32 | 2 6 3 | 2 1 6 |
| 33 | 2 9 9 | 2 4 11 |
| 34 | 2 13 9 | 2 8 9 |
| 35 | 2 18 3 | 2 13 3 |
| 36 | 3 3 5 | 2 18 3 |
| 37 | 3 9 5 | 3 4 2 |
| 38 | 3 16 4 | 3 11 1 |
| 39 | 4 4 7 | 3 19 4 |
| 40 | 4 14 6 | 4 9 2 |

Note.—Anyone may enter under above Table for any pension they may desire. The above Pensions are exlusive of bonus additions, which it is hoped will materially increase the payments to Pensioners when they come on the Fund.

### VI.—Pensions only by one Payment (returnable or not returnable)

Showing the Single Payment to secure a Pension of £10 per annum, to commence at a given age.

FOR MATRONS, SISTERS, AND NURSES.

| Age next birthday. | Pension to commence at age 50. | | Pension to commence at age 55. | | Pension to commence at age 60. | |
|---|---|---|---|---|---|---|
| | Contribution returnable on death or withdrawal. | Contribution not returnable. | Contribution returnable on death or withdrawal. | Contribution not returnable. | Contribution returnable on death or withdrawal. | Contribution not returnable. |
| | £ s. d. | £ s. d. | £ s. d. | £ s. d. | £ s. d. | £ s. d. |
| 20 | 71 15 0 | 53 16 8 | 55 0 0 | 38 4 2 | 41 0 10 | 25 17 6 |
| 21 | 73 18 4 | 55 16 8 | 56 13 4 | 39 13 4 | 42 5 10 | 26 16 8 |
| 22 | 76 2 6 | 57 19 2 | 58 7 6 | 41 2 6 | 43 11 8 | 27 16 8 |
| 23 | 78 8 4 | 60 3 4 | 60 2 6 | 42 14 2 | 45 17 6 | 28 18 4 |
| 24 | 80 15 0 | 62 9 2 | 61 18 4 | 44 6 8 | 46 4 2 | 30 0 0 |
| 25 | 83 4 2 | 64 16 8 | 63 15 0 | 46 0 10 | 47 12 6 | 31 2 6 |
| 26 | 85 13 4 | 67 6 8 | 65 13 4 | 47 15 10 | 49 0 10 | 32 6 8 |
| 27 | 88 5 0 | 69 19 2 | 67 13 4 | 49 13 4 | 50 10 0 | 33 11 8 |
| 28 | 90 18 4 | 72 13 4 | 69 13 4 | 51 11 8 | 52 0 10 | 34 18 4 |
| 29 | 93 12 6 | 75 9 2 | 71 15 0 | 53 11 8 | 53 10 10 | 36 5 0 |
| 30 | 96 9 2 | 78 9 2 | 73 18 4 | 55 13 4 | 55 4 2 | 37 3 4 |
| 31 | 99 6 8 | 81 10 10 | 76 2 6 | 57 17 6 | 56 17 6 | 39 3 4 |
| 32 | 102 5 10 | 84 15 0 | 78 8 4 | 60 3 4 | 58 10 10 | 40 14 2 |
| 33 | 105 7 6 | 88 2 6 | 80 15 0 | 62 11 8 | 60 6 8 | 42 6 8 |
| 34 | 108 10 10 | 91 13 4 | 83 4 2 | 65 1 8 | 62 2 6 | 44 0 10 |
| 35 | 111 15 10 | 95 6 8 | 85 14 2 | 67 13 4 | 63 19 2 | 45 15 10 |
| 36 | 115 2 6 | 99 3 4 | 88 5 0 | 70 5 4 | 65 18 4 | 47 12 6 |
| 37 | 118 12 6 | 103 4 2 | 90 10 4 | 73 5 0 | 67 7 6 | 49 11 8 |
| 38 | 122 3 4 | 107 8 4 | 93 12 6 | 76 5 0 | 69 18 4 | 51 11 8 |
| 39 | 125 16 8 | 111 16 8 | 96 9 2 | 79 7 6 | 72 0 0 | 53 14 2 |
| 40 | 129 12 6 | 116 8 4 | 99 6 8 | 82 12 6 | 74 3 4 | 55 18 4 |
| 41 | | | 102 6 8 | 86 1 8 | 76 8 4 | 58 5 0 |
| 42 | | | 105 7 6 | 89 13 4 | 78 14 2 | 60 13 4 |
| 43 | | | 108 10 10 | 93 8 4 | 81 0 10 | 63 4 2 |
| 44 | | | 111 15 10 | 97 7 6 | 83 10 0 | 65 17 6 |
| 45 | | | 115 3 4 | 101 10 0 | 86 0 0 | 68 13 4 |
| 46 | | | | | 88 11 8 | 71 11 8 |
| 47 | | | | | 91 4 2 | 74 13 4 |
| 48 | | | | | 93 19 2 | 77 18 4 |
| 49 | | | | | 96 15 10 | 81 6 8 |
| 50 | | | | | 99 14 2 | 84 19 2 |

Note.—Anyone may enter under the above table for any pension they may desire. Thus, if a sister or nurse wished to secure a pension of £50 per annum, she would have to pay five times the sum entered in the table opposite her age next birthday. If one of £20, twice the rates; if of £25, two-and-a-half times the rates, and so on.

### The National Pension Fund: How to Use It.

It may be taken for granted that no man or woman, whether old or young, rich or poor, has any objection to an honourable pension. From time immemorial pensions of all kinds have been favourite forms of rewarding merit; and so highly has the reward in many instances been appreciated, that even among some of the wealthiest and most illustrious families pensions have descended from father to son, from son to grandson, until finally they have become, as in many instances they were intended to become, "perpetual."

A "pension," a secure weekly allowance, which is as regular as the days of the week, is, to any one unable to work, either from sickness or approaching age, a kind of material "salvation." The most urgent of all needs is the need for daily bread; the most heartbreaking of all anxieties is anxiety on this behalf. A man or a woman will fight through almost any difficulty, so long as the means of living are secure. But when, in addition to a thousand other cares, there is the feeling that something unforeseen may happen which will leave the struggler penniless and destitute, such a feeling makes the stoutest heart quail, and the proudest spirit fearful.

Sick nurses, as a rule, do not belong to the wealthier classes. Even those among them who are ladies by birth and breeding are often entirely dependent on their own exertions for a livelihood. To such a class provision against sickness and old age makes all the difference between a life of cheerful contentment and hope, and a life of dull aching care and hopeless anxiety about sickness and advancing years.

The National Pension Fund for Nurses and Hospital Officials has been instituted, and is now in operation at its central offices, 38, Old Jewry, Cheapside. It is in the first place a fund which will be provided for and maintained by the beneficiaries themselves. But it is more than that, it is a fund which will be largely added to and made a reward of merit by those who have the interests of nurses and sick nursing at heart. Already there is a thousand a year available for distribution among those who may subscribe for pensions. If in such a short time so large a sum as a thousand a year is secured for addition to pensions, how much more may be expected in twenty years—that is before many pensions fall due?

With such a commencement thousands of nurses may be expected to be now considering the question whether or not they shall make an effort to join the Fund; and, in the event of their joining it, what particular table of pension and sick insurance they will choose. They must remember that they cannot both "eat their cake and have it." If they spend all the salary they now receive they cannot lay by for a rainy day, and so there is an end of the matter. But if they are willing to cut their cake into seven or eight equal portions, and put away one of these annually, that portion will grow bigger each year by the addition of interest raised upon it, and not only so, but will be added to by a definite proportion of the thousand a year already available, and other sums which will be contributed in future by other generous friends of nurses and nursing. It may be expected that the yearly pension which each nurse's own annual contributions would produce, will be almost, and perhaps quite, doubled by the added bonuses which have already been so generously commenced. So that a nurse whose premium of £3 5*s.* per annum would entitle her to a pension of £15 may expect, when the pension finally falls due, to receive nearly £30 a-year, or a little over 11*s.* 6*d.* per week.

That is the broad view which may be taken of the National Pension Fund as a whole. But the circumstances of nurses differ, and so there ought to be, and are, five or six different methods offered by the Fund of providing for sickness or old age, or both. These methods are clearly set forth in the foregoing tables. Many nurses, as well as other people, do not understand insurance and pension tables. Where there is any uncertainty two or three should meet together and try to master the different tables, so as to find out which would best meet their own individual cases. If their united wisdom should fail, they may seek the aid of the matron; and if, as will sometimes happen, the matron herself should see unfathomable mysteries, the secretary —always a man of figures—may be called in to illuminate the darkness.

Each table, it should be noted, has an explanatory heading, and if that be thoroughly comprehended all the rest is simple. For example, Table I. provides for pensions at the ages of 50, 55, or 60. According to this table the premiums paid by nurses are not returnable except as pensions. But then, as a compensation for that, the premiums paid yearly are low, and the pensions to be received are high. The nurse who cannot afford a high premium, and who has little hope of marriage or a fortune, should choose this table because of the large returns she will receive as pension when age approaches. The pensions under this table, secured by nurses' premiums, amount to £15 a-year at 50 years of age, £22 10*s.* at 55, and £30 at 60. But with the added bonuses these amounts may be nearly doubled, so that the nurse who can continue to work until she is 60 may expect at least £52 a-year, or a pound a week for the remainder of her life.

According to Table II., the premiums paid are returnable under certain conditions. Some who are uncertain whether or not they will continue to devote themselves to nursing, or who may "have expectations," will find this more suitable than Table I.

These two are given as examples of the whole. It is not necessary to enter more into detail. A little careful study will make the matter quite plain. What it is desirable to repeat is that this Fund, if taken advantage of by nurses, will place them in an absolutely secure and relatively more advantageous position than is enjoyed by any of the learned professions, or by persons whose living depends upon the pursuit of trade. When once a nurse is accepted, and has paid her first premium, she is forthwith entitled to sick pay according to a fixed and liberal scale; she is entitled to a sufficient retiring pension, if permanently disabled by sickness or accident; and to a life pension, when the limit of age—50, 55, or 60—is reached. Under this scheme charity is for ever unnecessary, and want is rendered impossible. The fund is frankly commended to all who give themselves to the duty of nursing the sick, as a means of relieving them from the crushing burden of pecuniary anxiety, and enabling them to devote themselves with whole-hearted service to their great and noble work.

